# Validity of Nutrient Intakes Derived from an Internet Website Dish-Based Dietary Record for Self-Management of Weight among Japanese Women

**DOI:** 10.3390/nu9101058

**Published:** 2017-09-24

**Authors:** Eri Matsuzaki, Mikiko Michie, Terue Kawabata

**Affiliations:** 1 Faculty of Nutrition, Kagawa Nutrition University, 3-9-21 Chiyoda, Sakado, Saitama 350-0288, Japan; kawabata@eiyo.ac.jp; 2 Asken division, Green House Co., Ltd., 3-20-2 Nishishinjyuku, Shinjyuku-ku, Tokyo 163-1477, Japan; mikiko-michie@greenhouse.co.jp

**Keywords:** web, Internet, validity, nutrient intakes, dish-based dietary record

## Abstract

We compared the relative validity of nutrient intakes derived from Internet website dish-based dietary records (WDDRs) and weighed dietary records (DRs). The study participants were 218 registered users of a dietary management website. After excluding 55 participants, a total of 163 participants were included in the final analyses. The participants were Japanese women, and their mean age and mean body mass index (BMI) were 39.3 (±10.3) years and 22.3 (±3.7) years, respectively. We compared differences in the DR and WDDR with respect to intakes of energy and 13 nutrients. The median values for the intake of protein and seven nutrients showed no significant difference between the DR and WDDR, and the estimated values were generally similar. The interquartile range of nutrients with a significant difference did not show a large deviation, with the exception of vitamin A. Correlation coefficients showed a strong correlation of 0.7, with the exception of sodium and vitamin E. The kappa coefficients for energy and for some nutrients were good to fair. Using Bland–Altman plots, we found proportional errors in the intake values for vitamins and minerals. We could not confirm obvious systematic errors for energy, protein, fat, or carbohydrate intake. WDDR use is considered to be a valid method for estimating energy and nutrient intakes among Japanese women.

## 1. Introduction

In recent years, about 60% of deaths in Japan are due to cancer, heart disease, and stroke [1], which are caused by obesity, hypertension, dyslipidemia, and hyperglycemia [2]. Visceral fat-type obesity is one of the factors of lifestyle-related diseases that should be improved first, because it is the basis of metabolic syndrome [3]. The proportion of obesity in the Japanese population over the past 10 years has been around 30% for men and more than 20% for middle-aged and older women, although a downward trend has been seen in women [4]. In addition, the proportion of people over 40 years of age who are suspected of having metabolic syndrome is 48.3% for men and 20.6% for women [4]. In order to prevent the onset of lifestyle-related disease conditions, it is important to self-manage diet and exercise daily. However, it is difficult to fully understand how to maintain a balanced diet and to practice moderate exercise in daily life; moreover, it is hard to remain motivated to continue these habits over the long term.

To solve this problem, various programs that help the general public to continuously practice proper eating and exercise habits have been developed [5]. In particular, the number of weight-loss and lifestyle-improvement methods using the Internet has been rapidly increasing in recent years [6,7,8,9]. This study examined users of Asken, an Internet website that generates and records dish-based dietary records [10] and that is intended for Japanese users. However, an application of the same tool is available in English in Canada [11], with dishes that are commonly eaten in Canada and the United States. There are plans underway to release the application in the United States in the future.

In Japan, Asken is used not only by experts, but also by the general public for the main purpose of self-management of weight. As of March 2015, this website had more than 400,000 registered users, making it one of the largest websites for dietary management in Japan [10]. The website instantly estimates the daily energy and nutrient intakes of website users on the basis of the Dietary Reference Intakes for Japanese 2015 [12]. The range of the recommended intake is visualized with a graph, and individualized dietary advice is provided. The 663 users who continuously used this website for 3 months showed dietary improvement and an average weight reduction of 3.5 kg [13].

**Figure 1:**
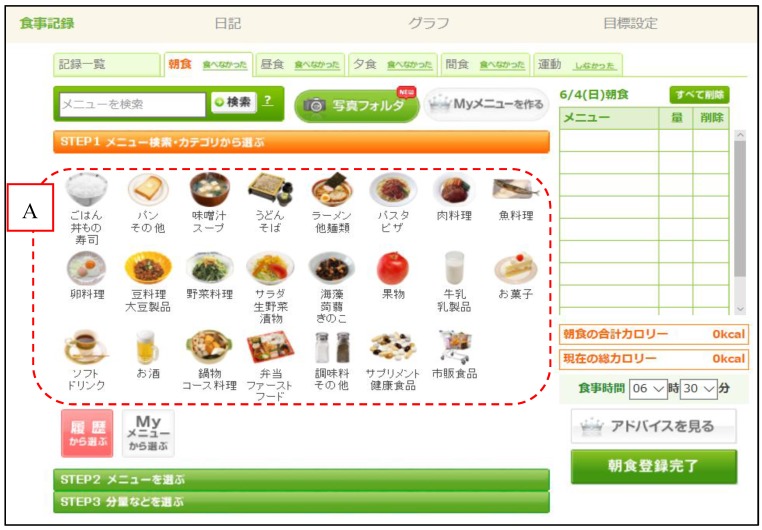
Creating a dish record using the Japanese version of Asken. Users select the category of their dish and then choose the specific dish. Translation of dish names in area A (left to right): Top row: rice, bread, miso soup, soba, ramen, pasta, meat dishes, and fish dishes. Middle row: egg dishes, bean dishes, vegetable dishes, salads, seaweed and mushrooms, fruits, milk, and confectioneries. Bottom row: beverages, alcoholic beverages, three-course meals, boxed lunches and fast food, seasoning, supplements, and ready-made food.

**Figure 2:**
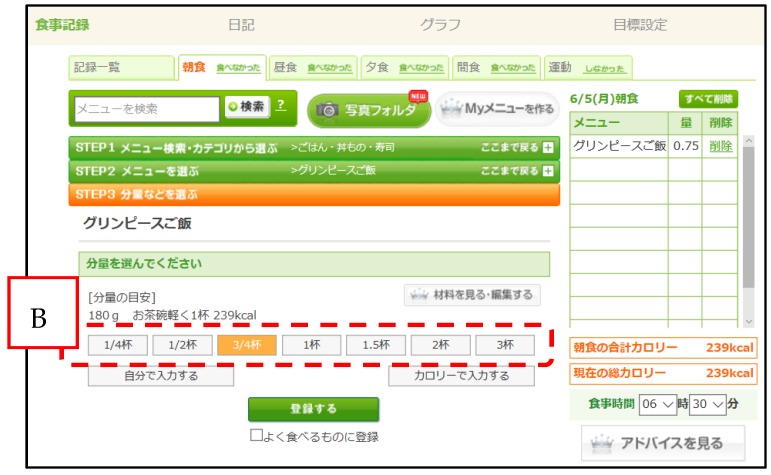
Choosing the serving size for a dish using the Japanese version of Asken. After selecting the specific dish, users select the appropriate serving size for the dish they consumed. Translation of serving sizes in area B (left to right): 1/4 of the serving, 1/2 of the serving, 3/4 of the serving, 1 serving, 1.5 servings, 2 servings, and 3 servings.

**Figure 3:**
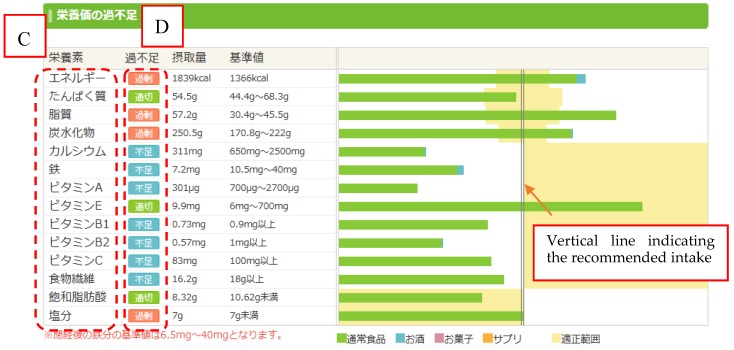
Graph of energy and nutrient intakes generated by Asken on the basis of data input by the user. Translation of text in area C (top to bottom): energy, protein, lipids, carbohydrates, calcium, iron, vitamin A, vitamin E, vitamin B1, vitamin B2, vitamin C, dietary fiber, saturated fatty acids (not included in the present study), and salt. In area D, red boxes indicate excessive intakes, green boxes indicate appropriate intakes, and blue boxes indicate deficiencies. The vertical line shows the recommended intake.

Methods for accurately evaluating energy and nutrient intakes include dietary record (DR) methods (e.g., weighed record method, estimated record method) and dietary recall methods. Recording all food items and their weight is time-consuming and a great deal of work, and the calculation and assessment of nutrient intakes requires the support of experts such as dietitians. To overcome these challenges, Asken uses an input method based on the dish consumed. The user logs into the site, selects their meal from an online database containing approximately 100,000 dishes, and inputs the serving size they consumed (Figure 1 and Figure 2). Following this, the user can check graphs of energy and nutrient intakes that are generated on the basis of the data they entered (Figure 3). Although this method is simple, the ingredients and weight of each registered dish on the website are fixed. Even when users cook their own meals, they cannot make adjustments to the dishes registered in Asken to exactly match what they prepared. Therefore, even if the names of the dishes match, there is a possibility that the food items consumed do not match the contents of the dish selected from the database exactly.

Although previous studies have investigated the validity of dietary survey methods, there are few studies on the validity of dish-based dietary survey methods. Furthermore, the authors found no study on the validity of DR tools that are comparable to the Asken website in terms of the number of dishes in the database. Therefore, in this study, we performed an objective evaluation of the validity of an Internet website dish-based DR (WDDR) tool. The objectives of this study were to separately examine the ability of the WDDR to estimate representative nutrient intake values at the population level, that is, for all the participants of this study (intake estimating ability), to rank the intake of individuals within the population (ranking ability), and to estimate intake at the individual level.

## 2. Materials and Methods

### 2.1. Participants and Investigation Period

Study participants were recruited from among registered users of the Japanese version of Asken using Internet mail (email magazines). The first survey was carried out from July to August 2013 and the second was carried out in May 2014.

Email magazines are sent periodically to registered users of Asken to provide them with health and diet information. Healthy persons over 20 years of age were eligible to participate in the study, regardless of gender. However, only women were recruited in 2014, because there were few responses from men in 2013. After the recruitment email was sent, we set an “application period” of 7 days, followed by a “preparation period” of 7 days, and a “study period” of 7 days. During the application period, we obtained responses from 100 participants (14 men and 86 women; average age of 42.6 years) in 2013 and 118 participants (all women; average age of 37.4 years) in 2014. A total of 218 participants (14 men and 204 women; average age of 39.9 years) completed the surveys conducted in 2013 and 2014, and 55 participants were excluded from this study. The reasons for exclusion were gender (male participants: *n* = 14) and insufficient DRs, such as those missing photographs or other information (*n* = 41). As a result, data for a total of 163 participants were analyzed in this study. The participants recorded all food items on the DR and the WDDR, and we compared the energy and nutrient intakes obtained by the DR with those obtained by the WDDR.

Prior to beginning the study, the participants provided consent to participate by clicking the “agree” button displayed on their screen at the time of registration after accepting the terms of use and confirming that they understood the explanations for the handling of personal information. This study was conducted in accordance with the Declaration of Helsinki, and the protocol was approved by the Kagawa Nutrition University ethics review committee (No. 223/2013). 

### 2.2. DR

An explanation of the study purpose, study methods, and an example of a completed DR form were attached to an email and sent to all the study participants. The date of the dietary survey was set to one weekday during the study period. All foods consumed between getting up in the morning and going to bed at night, including breakfast, lunch, dinner, and snacks, were recorded on the specified DR form. In addition, the participants were required to take a photograph of their meal before eating and to store the digital images separately from the DR. The DR form was sent by email attachment or postal mail, and the digital images of the dishes were returned by uploading the files on a special screen. We used the digital images to confirm the contents of each meal by comparing the photograph of each dish with the DR completed by the participant. When there were discrepancies, the contents of the DR were modified to more closely match the digital image.

The methods for completing the DR were as follows. When weighing the food items at home, in principle, all ingredients and seasonings were weighed in their raw state before cooking. In cases when a food could not be weighed in its raw state, it was weighed by separating each food ingredient after cooking, and the weight of each was recorded along with a statement of “cooked” on the DR form. For processed foods, a product name, a company name, and the amount of intake (such as half of one box or one package) were recorded. The participants used their own scale for measuring, and weights were recorded in grams. In the case of eating out, the ingredients and the estimated serving (such as one large bowl or one small plate) of the dish along with the name of the restaurant and the dish were recorded. When the full dish was not eaten, the estimated leftover portion was subtracted from the total serving and the modification was made on the DR form. The participants took the photographs with their own digital camera or cell-phone camera. After we received the DR, we confirmed the food names and weights recorded on the DR form by comparing them with the participants’ photographs, as described above. For cooked foods, we calculated nutrients on the basis of the weight of the food in its raw state. For processed foods, we analyzed the ingredients in accordance with the nutrition facts indicated by the manufacturer. Lastly, nutrient values of dietary supplements were not included in the nutrient intakes for this study. Nutrient intakes were calculated using the Standard Tables of Food Composition in Japan 2010 [14].

### 2.3. WDDR

The WDDR was completed on the same day as the DR. The participants were asked to enter the data on the website in the same way as they usually did. When recording food intakes on the website, users choose their portion size from one of the seven options: 1/4 of the serving, 1/2 of the serving, 3/4 of the serving, 1 serving, 1.5 servings, 2 servings, or 3 servings. The nutrient intake recorded on the WDDR was automatically calculated by the website system on the basis of the data provided by the participant. The system calculated the nutrient intake on the basis of the weight before cooking using the Standard Tables of Food Composition in Japan 2010 [15]. For processed foods, the nutrient content data provided by the manufacturer were used.

### 2.4. Nutrient Intake Analysis

The nutrients analyzed and evaluated in this study were the 13 nutrients listed on Asken (protein, fat, carbohydrates, calcium, iron, vitamin A, vitamin B1, vitamin B2, vitamin C, vitamin E, dietary fiber, cholesterol, and sodium) and energy.

### 2.5. Statistical Analysis

Statistical analysis was performed using JMP version 10 (SAS Institute Inc., Cary, NC, USA). JMP can calculate kappa coefficients but does not calculate weighted kappa coefficients; therefore, we used Excel Statistics, 2012 edition (Social Survey Research Information Co., Ltd., Tokyo, Japan) for the weighted kappa coefficients. The energy and nutrient intakes for the DR and the WDDR are presented as medians and quartile ranges. Comparisons between the DR and WDDR were conducted using the Wilcoxon signed-rank test. The relationship between the DR and WDDR was examined by Spearman’s rank correlation, and *p*-values less than 0.05 were considered statistically significant. The degree of agreement between individual nutrient intakes was tested using weighted kappa coefficients [16] and Bland–Altman plots [15]. For the weighted kappa coefficients, linear weights were calculated, following previous studies [17,18]. The limit of agreement, which indicates the limit of significant difference in the Bland–Altman plot, was set to 1.96 times the standard deviation of the difference between the DR and WDDR [19]. The evaluation criteria for the correlation coefficients were as follows: 0–0.19: almost no correlation; 0.20–0.39: weak correlation; 0.40–0.69: moderate correlation; and 0.70–1.00: strong correlation [20]. The evaluation criteria for weighted kappa coefficients were as follows: 0–0.40: poor agreement; 0.41–0.60: moderate agreement; 0.61–0.80: good agreement; and 0.81–1.00: very good agreement [21]. The Wilcoxon signed-rank test used both unadjusted values and adjusted values of the density method. Spearman’s rank correlation, the weighted kappa coefficients and Bland–Altman plots used only adjusted values of the density method. For the density method, the amount of nutrient intake was converted into intake (mg or μg) per 1000 kcal.

## 3. Results

### 3.1. Participant Attributes

The characteristics of the participants are shown in Table 1. The average age of the participants was 39.3 years, and the average body mass index was 22.3. Of the 163 participants, 10 completed the survey for 2 days and 2 completed the survey for 3 days. Because these data were registered in a different time period, each entry was included in the analysis as a record for one survey.

### 3.2. Comparison and Correlation of the DR and WDDR

The comparisons and correlations between the DR and WDDR for energy and nutrient intakes are shown in Table 2. The median energy intake was significantly higher in the WDDR (1554 kcal/day) compared to the DR (1472 kcal/day). Similarly, carbohydrates, vitamin A, and sodium were significantly higher in the WDDR compared to the DR, while fat, vitamin C, and cholesterol were significantly lower. No significant difference was seen between the WDDR and DR for protein, calcium, iron, vitamin B1, vitamin B2, vitamin E, or dietary fiber. Spearman’s correlation coefficient after the energy adjustment showed a moderate association between the intakes of vitamin E, at 0.62, and sodium, at 0.49; the intakes of all other nutrients showed a strong association between the DR and WDDR.

### 3.3. Agreement between the DR and the WDDR

Table 3 shows the agreement between the DR and the WDDR on the basis of the quantile and weighted kappa coefficients by the energy and nutrient intake. For the energy intake, 66.3% were divided into the same quantile, showing good agreement between the DR and the WDDR, with a weighted kappa coefficient of 0.70. Nutrients that showed good agreement between the DR and the WDDR were calcium, iron, vitamin C, and dietary fiber. Sodium intake showed poor agreement between the DR and the WDDR, with a weighted kappa coefficient of 0.34. Other nutrients showed moderate agreement. Except for vitamin B1 (49.1%) and sodium (41.1%), agreement was observed for over 50% of the participants for all the other nutrients, and all were divided into the same quantile. More than 81% of all the nutrients surveyed were divided into the adjacent quantile. The proportion of the most distant quantile was 0% for energy, protein, carbohydrates, iron, vitamin B1, and vitamin B2. Vitamin E, with a proportion of 2.5%, and sodium, with a proportion of 4.3%, were in the most distant quantile.

### 3.4. Agreement between the DR and the WDDR by Bland–Altman Plot

**Figure 4:**
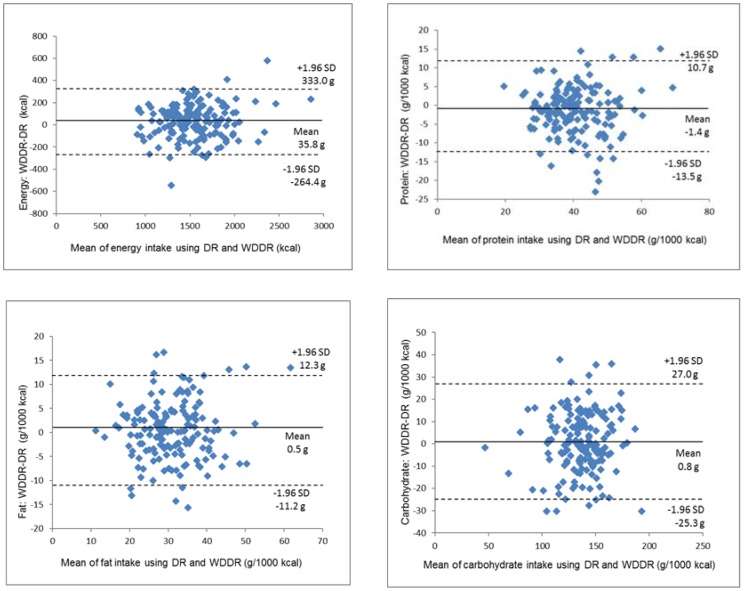
Bland–Altman plots for agreement between energy and energy-adjusted intakes of nutrients (protein, fat and carbohydrates) using the DR and WDDR for 163 women for 1 day. DR: dietary record; WDDR: website dish-based dietary record; SD: standard deviation.

**Figure 5:**
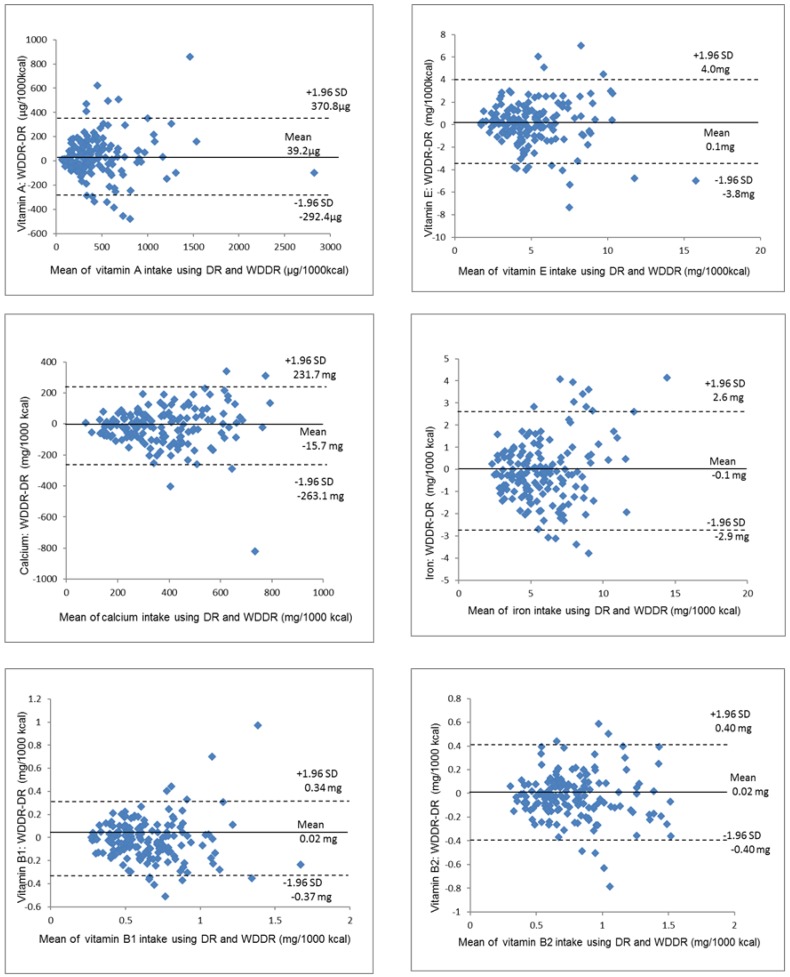
Bland–Altman plots for agreement between energy-adjusted intakes of nutrients (calcium, iron, vitamin A, vitamin E, vitamin B1 and vitamin B2) using the DR and WDDR for 163 women for 1 day. DR: dietary record; WDDR: website dish-based dietary record; SD: standard deviation.

**Figure 6:**
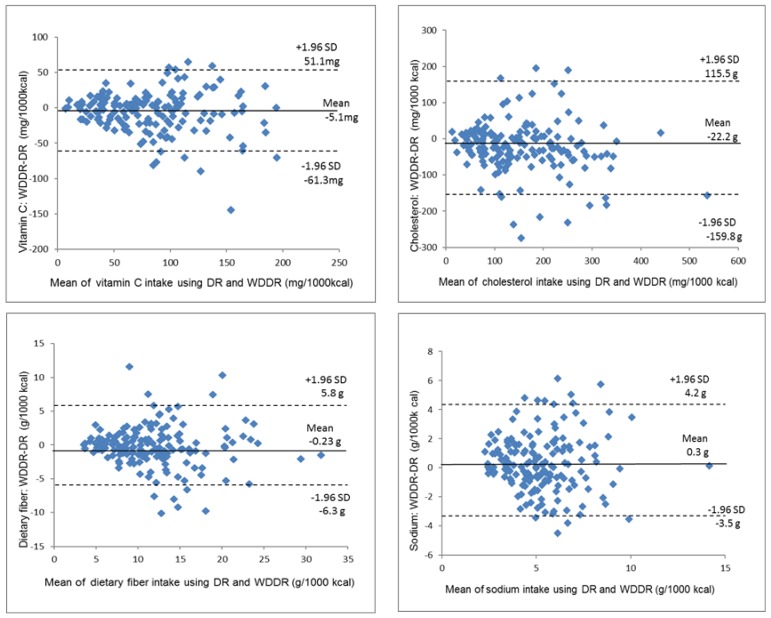
Bland–Altman plots for agreement between energy-adjusted intakes of nutrients (vitamin C, cholesterol, dietary fiber and sodium) using the DR and WDDR for 163 women for 1 day. DR: dietary record; WDDR: website dish-based dietary record; SD: standard deviation.

Figure 4, Figure 5 and Figure 6 show the Bland–Altman plots of the energy and nutrient intake. For the energy, protein, fat, and carbohydrate intake, obvious systematic errors could not be confirmed. The range of significant differences for energy, protein and carbohydrates were between −261.4 and 333.0 kcal, −13.5 and 10.7 g, and −25.3 and 27.0 g, respectively. These values from the WDDR were one-third to around one-half of the mean DR values, and the average energy intake on the WDDR was 35.8 kcal higher than that of the DR. Differences in the vitamin and mineral (vitamin A, vitamin B1, vitamin B2, vitamin C, vitamin E, calcium, iron and sodium) intake and dietary fiber increased as the average value increased, showing a tendency for proportional bias.

## 4. Discussion

In this study, we examined the validity of the WDDR method, which is easy for the general public to use, compared to the weighed DR method. We also examined representative nutrient intake values at the population level, the intake ranking ability of individuals within the population, and the intake estimating ability at the individual level among Japanese women. In comparing the representative nutrient intake values of 13 nutrients and energy, we did not observe significant differences in the values of 7 nutrients or energy. In addition, the median representative nutrient intake values were approximately the same for the DR and WDDR. Therefore, the WDDR was considered useful for estimating nutrient intakes at the population level among Japanese women.

When evaluating the validity of nutrient intakes, a good correlation coefficient between the DR and the food frequency questionnaire (FFQ) is generally in the range of 0.5–0.7 [22]. A study using the FFQ conducted in the United Kingdom [23] found correlation coefficients of 0.23–0.65, which were approximately equal to those found in other studies [24,25]. The validity of dietary surveys in the Japanese population is generally lower than that in Western populations, because many Japanese dishes are a combination of various food items, such as boiled and marinated dishes containing multiple ingredients [26]. A previous study in Japan reported that a correlation coefficient of 0.4 or higher has a reasonable ranking ability [19]. The correlation coefficient in this study was 0.49–0.84; therefore, the WDDR is considered to be a useful method for ranking the nutrient intake of individuals within the Japanese population.

This study investigated the agreement of nutrient intakes between two survey methods using the weighted kappa coefficient. In a previous study comparing 24 h dietary recalls and the FFQ in Chile [18], the kappa coefficients of all nutrient intakes, including energy, were less than 0.47. Compared to the kappa coefficients in studies on the validity of the FFQ, the degree of agreement between the WDDR and the DR for nutrient intakes in this study was relatively high, with the exception of sodium. However, because the degree of agreement for the sodium intake between the WDDR and the DR was low in this study, it is considered difficult to estimate sodium intake by evaluation at the individual level.

In Bland–Altman plots, a greater range of significant difference means a larger error, but the allowable range of significant difference depends on the study [27,28]. Another study comparing the FFQ and the DR in Mexico [29] found that the average energy intake according to the DR was 2041 kcal, and the range of significant difference was between −1070 and 2190 kcal, which was larger than for our study. Furthermore, another study evaluating individual nutrient intakes in the Japanese population using a Bland–Altman plot examined the estimation accuracy of the energy intake according to the weighed DR using the double-labeled water method [30] and found that the range of significant difference for energy intake in women was between −1396 and 952 kcal/day, which was greater than for our study. Our study cannot be simply compared with most previous studies on the validity of the FFQ, but the energy intake errors for individuals using the WDDR in this study were relatively small. Additionally, regarding the protein, fat, and carbohydrate intakes, statistical errors were small compared to other studies using the Bland–Altman plot [23,31]. This suggests that website users can effectively use the WDDR to estimate their energy, protein, fat and carbohydrate intake.

On the other hand, in terms of micronutrients, systematic errors, whose difference becomes larger as the intake increases, were observed for many of the nutrients in the Bland–Altman plot. As a result, the degree of agreement in the intake estimation at the individual level may have varied depending on the amount of intake. In addition, both the correlation coefficient and the kappa coefficient of sodium were lower than those of the other nutrients evaluated. Although the website includes the standard amount of seasoning for each dish registered in the database, the actual amount of seasoning in the dish varies depending on individual preferences. Thus, inconsistencies between what is considered a single serving by users and the predetermined serving size on the website could be the main reason for the low agreement between the DR and the WDDR for sodium. 

The WDDR aims to more accurately estimate the amount of nutrient intake per day on the basis of dish-based records. Continuing this for some number of days is considered to lead to a more accurate estimation of habitual nutrient intake.

## 5. Limitations

The objective of this study was to examine the validity of nutrient intakes as estimated by an Internet WDDR tool. The WDDR was completed on the same day as the DR in this study. Because of the educative effects, it is possible that the participants entered more accurate information about the meals they consumed on the website. Additionally, women tend to cook meals more often than men, and this may have influenced the accuracy of the records [32]. The participants of this study were relatively young women with an average age of 39.3 years, and they may have been accustomed to using websites. This may have contributed to the favorable results. Although we initially recruited both men and women for the survey, only women were included in the analysis because of a shortage of male participants. Cases of gender differences between men and women have been noted in some dietary studies [22,33]. For this reason, in order to confirm the validity of the WDDR, a similar study that includes men should be conducted in the future.

We compared the WDDR with the DR and considered its relative validity. To the best of our knowledge, no research on the validity of dish-based DRs is available; therefore, we compared the results of the WDDR with the results of other methods. We often examine the validity of other methods by comparing them with the DR. For this reason, it was considered possible to compare the results of the WDDR with those of other methods.

## 6. Conclusions

On the basis of the results of the present study, the WDDR is a useful method for estimating energy and nutrient intakes at the population level (i.e., for all the participants in the present study) and can rank the intake of individuals within the population with a good degree of accuracy for Japanese women. The WDDR showed a relatively high estimating accuracy for the energy intake among individuals. In particular, it showed a relatively high estimating accuracy for the fat, protein and carbohydrate intakes among individuals. However, proportional error should be considered when evaluating the intakes of vitamins and minerals. In addition, it was difficult to accurately estimate the sodium intake with the WDDR. However, on the basis of our results, the WDDR is considered to be a valid method for estimating the energy and nutrient intake among Japanese women.

## Figures and Tables

**Table 1:** Participant characteristics ^1^.

	Women (*n* = 163)		
	*n* (%)	Mean	SD ^2^
Age (years)	-	39.3	±10.3
Height (cm)	-	158.4	±5.6
Body mass index (kg/m^2^)	-	22.3	±3.7
Accustomed to using Asken	-	-	-
Yes	94 (57.7)	-	-
No	69 (42.3)	-	-
Physical activity	-	-	-
Inactive	94 (57.7)	-	-
Moderate	64 (39.3)	-	-
Active	7 (4.3)	-	-
Employment status	-	-	-
Manager	2 (1.2)	-	-
Employee	40 (13.5)	-	-
Freelancer	22 (18.4)	-	-
Part-timer	30 (30.1)	-	-
Housewife	49 (4.9)	-	-
Student	8 (4.9)	-	-
Other	12 (7.4)	-	-

^1^ Based on self-reported characteristics. ^2^ SD: standard deviation.

**Table 2:** Comparison and correlation of the DR and WDDR by energy and nutrient intake (*n* = 163).

	DR		WDDR			Spearman’s Correlation Coefficient		
	Median ^1^	Inter-Quartile Range	Median ^1^	Inter-Quartile Range	Test ^2^	*r*	*r* (Energy-Adjusted)	Evaluation
Energy (kcal/day)	1472	1307–1716	1554	1359–1732	**	0.87	—	Strong
Protein (g/day)	61.6	50.0–72.0	61.3	49.9–69.2		0.78	0.75	Strong
Fat (g/day)	45.9	34.8–55.5	45.7	36.6–60.1	**	0.75	0.72	Strong
Carbohydrates (g/day)	208.1	173.4–236.3	215.6	187.6–245.2	*	0.82	0.79	Strong
Calcium (mg/day)	529	394–728	533	392–666		0.77	0.79	Strong
Iron (mg/day)	8.2	6.4–10.7	8.2	6.3–10.3		0.81	0.84	Strong
Vitamin A (μg/day)	498	305–797	571	349–917	***	0.78	0.78	Strong
Vitamin B1 (mg/day)	0.92	0.67–1.22	0.92	0.68–1.15		0.76	0.77	Strong
Vitamin B2 (mg/day)	1.13	0.92–1.41	1.17	0.91–1.36		0.71	0.75	Strong
Vitamin C (mg/day)	124	85–163	117	77–155	*	0.81	0.82	Strong
Vitamin E (mg/day)	6.9	5.0–9.6	7.2	5.0–10.0		0.66	0.62	Moderate
Cholesterol (mg/day)	222	123–351	193	111–310	***	0.73	0.71	Strong
Dietary fiber (g/day)	16.6	12.0–21.7	16.4	12.0–21.5		0.79	0.81	Strong
Sodium (g/day)	7.3	5.1–10.0	7.7	6.0–10.0	*	0.59	0.49	Moderate

DR: dietary record; WDDR: website dish-based dietary record. ^1^ Unadjusted value; ^2^ Wilcoxon signed-rank test. * *p* < 0.05; ** *p* < 0.01; *** *p* < 0.001.

**Table 3:** Agreement between the DR and WDDR by energy and nutrient intake ^1^.

	Quantile					Weighted Kappa Coefficient		
	Same Quantile (%) ^1^	Adjacent Quantile (%) ^1^	±2 (%)	±3 (%)	a + b (%)	Kw	LOA	Evaluation
Energy	66.3	30.7	3.1	0.0	96.9	0.70	0.63–0.78	Good
Protein	51.5	38.0	10.4	0.0	89.6	0.53	0.44–0.62	Moderate
Fat	51.5	38.0	9.2	1.2	89.6	0.52	0.43–0.61	Moderate
Carbohydrates	50.9	41.7	7.4	0.0	92.6	0.55	0.46–0.63	Moderate
Calcium	57.1	37.4	4.9	0.6	94.5	0.61	0.52–0.69	Good
Iron	58.9	36.8	4.3	0.0	95.7	0.64	0.56–0.72	Good
Vitamin A	54.6	39.3	4.9	1.2	93.9	0.58	0.49–0.66	Moderate
Vitamin B1	49.1	42.9	8.0	0.0	92.0	0.53	0.44–0.61	Moderate
Vitamin B2	55.2	34.4	10.4	0.0	89.6	0.56	0.47–0.65	Moderate
Vitamin C	58.3	36.2	4.9	0.6	94.5	0.62	0.53–0.7	Good
Vitamin E	54.6	31.9	11.0	2.5	86.5	0.51	0.41–0.61	Moderate
Cholesterol	54.6	36.8	7.4	1.2	91.4	0.56	0.47–0.65	Moderate
Dietary fiber	62.6	31.3	4.3	1.8	93.9	0.64	0.55–0.72	Good
Sodium	41.1	39.9	14.7	4.3	81.0	0.34	0.23–0.45	Poor

DR: dietary record; WDDR: website dish-based dietary record; Kw: Linear weighted kappa coefficient; LOA: limit of agreement. ^1^ Energy-adjusted.
